# Donation after Circulatory Death in Paediatric Liver Transplantation: Current Status and Future Perspectives in the Machine Perfusion Era

**DOI:** 10.1155/2018/1756069

**Published:** 2018-03-18

**Authors:** Roberta Angelico, M. Thamara P. R. Perera, Tommaso Maria Manzia, Alessandro Parente, Chiara Grimaldi, Marco Spada

**Affiliations:** ^1^Division of Abdominal Transplantation and Hepatobiliopancreatic Surgery, Bambino Gesù Children's Research Hospital IRCCS, Rome, Italy; ^2^The Liver Unit, Queen Elizabeth Hospital, Birmingham B15 2TH, UK; ^3^Department of Experimental Medicine and Surgery, Liver Unit, Tor Vergata University of Rome, Rome, Italy

## Abstract

Efforts have been made by the transplant community to expand the deceased donor pool in paediatric liver transplantation (LT). The growing experience on donation after circulatory death (DCD) for adult LT has encouraged its use also in children, albeit in selective cases, opening new perspectives for paediatric patients. Even though there has recently been a slight increase in the number of DCD livers transplanted in children, with satisfactory graft and patient outcomes, the use of DCD grafts in paediatric recipients is still controversial due to morbid outcomes associated with DCD grafts. In this context, recent advances in the optimization of donor support by extracorporeal membrane oxygenation and in the graft preservation by liver machine perfusion could find application in order to expand the donor pool in paediatric LT. In the present study we review the current literature on DCD liver grafts transplanted in children and on the use of extracorporeal donor support and liver perfusion machines in paediatrics, with the aim of defining the current status and future perspectives of paediatric LT.

## 1. Introduction

Organ shortage is the main challenge in liver transplantation (LT) and the discrepancy between the number of waiting list patients and potential donor offers is greater in the paediatric field due to the scarcity of size-matched donors/grafts. The paediatric waiting list mortality rate nowadays is about 10%; however mortality appears to be highest in children younger than 6 years [1, 2]. Furthermore, in recent years a change in donor demographics has resulted in a reduction in the number of brain-dead donor livers suitable for splitting, which represent the major graft pool for children awaiting LT [2]. Consequently, alternative organ sources have been explored in order to increase organ availability for paediatric recipients by optimization of donor support, graft preservation, and surgical techniques.

In the last few decades, there has been a great interest in donation after circulatory death (DCD), the use of which has rapidly increased [3]. DCD donors are generally considered “extended-criteria donors.” In countries with an active DCD program, DCD donors account for 5% to 35% of the total donation [4, 5]. Hence, the use of DCD has significantly augmented the donor pool for LT [6]. Several studies have reported that patient survival after adult DCD LT is equivalent to that of donation after brain death (DBD), while graft survival is slightly inferior [7, 8]. Historically, the use of DCD grafts has been associated with higher risks of primary nonfunction (PNF), but the current incidence of PNF appears to be similar to that of DBD grafts due to better understanding and graft selection; but the problems surrounding DCD grafts such as ischaemic cholangiopathy, vascular thrombosis, and posttransplant acute kidney injury remain unsolved [9, 10]. Improved results with DCD have been obtained by avoiding high-risk factors related to the donor such as advanced donor age, overweight donor, prolonged cold ischemic time (CIT) and warm ischemic time (WIT), and recipient variables including retransplantation, need of intensive care at time of transplant, or renal dysfunction [11, 12]. Moreover, recent advances in improving graft quality, using donor extracorporeal support and liver machine perfusion, have further contributed to increased utilization and better results of DCD grafts [13–15].

Although the use of DCD livers in adult recipients is increasing, paediatric experience is limited. The use of DCD grafts in paediatric candidates for LT remains controversial and mainly single-center experiences have been reported. Nevertheless, paediatric DCD donors could constitute an important portion of the paediatric donor pool, since withdrawal of life-sustaining therapy accounts for 30–65% of deaths of patients in paediatric intensive therapy units (ITU) [16]. Moreover, as paediatric donors are mostly matched for paediatric recipients, the implementation of DCD in children could potentially reduce the paediatric waiting list mortality.

Recent advances in the optimization of donor stability with extracorporeal membrane oxygenation (ECMO) and the improvement of organ preservation with perfusion machine might also start a new era in graft availability for children, as these technological advances may help to minimize the morbid complications associated with conventional DCD grafts as well as to expand the safe utilization of extended-criteria DBD. Here we have reviewed the current literature on DCD liver grafts transplanted in children and the use of extracorporeal donor support and liver perfusion machine in paediatric donors. Our aim was to define the current status and future perspectives of paediatric LT.

## 2. Material and Methods

Two authors of this manuscript (R. A., A. P.) independently searched the PUBMED/MEDLINE database with the following key words:* paediatric transplantation, liver transplantation in children, donation after cardiac death, donation after circulatory death, non–heart-beating donors, liver splitting, partial liver graft, reduced liver graft, extracorporeal donor support, extracorporeal membrane oxygenation, organ preservation, normothermic regional perfusion, normothermic machine perfusion, hypothermic machine perfusion, *and* sub-normothermic machine perfusion*. All papers published in English between January 1995 and October 2017 and focusing on the following topics were retrieved: (1) donation after circulatory death used in paediatric LT (recipient age defined <18 years); (2) extracorporeal donor support used for paediatric LT; (3) liver perfusion machine used in paediatric LT; (4) splitting liver during perfusion machine. Studies of nonliver organs were excluded, as were studies where ECMO was used as a support for recipients awaiting LT or after transplantation. The selected papers included randomized controlled trials, conference consensus reports, meta-analyses, review articles, and pilot studies and case reports. Given the heterogeneity of the published papers and data, the present paper is in the form of a conventional review where important aspects of the aforementioned studies are highlighted.

We identified 48 relevant records on the use of DCD grafts in children, 41 of which were excluded as ineligible for the following reasons: 19 studies included non-LT from DCD donor to paediatric recipients, 18 reports focused on the ethical aspects and the implementation policy of paediatric DCD donation, and 4 studies included LT from paediatric DCD donor to adult recipients; the remaining 7 records, focusing on DCD grafts used in paediatric LT recipients, were included in the review [17–24].

Regarding the use of ECMO in paediatric LT, 19 records were identified, 17 of which were excluded because ECMO was used as bridge therapy while awaiting LT or as a support for complications after LT (i.e., sepsis, shock, and multiorgan failure). Therefore, only 2 records on the use of ECMO in liver donors for paediatric recipients were eligible [25, 26].

Out of 10 papers selected on the use of liver machine perfusion in human LT, none included paediatric recipients; therefore all of them were excluded. Finally, since split liver represents the most common type of graft used in paediatric LT, we considered 2 reports on the split liver procedure during machine perfusion, even if the grafts were not transplanted, in order to open new prospective on the machine perfusion applicability in children [27, 28].

## 3. The Use of Donation after Circulatory Death Grafts in Paediatric Liver Transplantation

Donation after circulatory death (DCD) involves donors with permanent absence of respiration and circulation, which have been defined by the Maastricht classification of uncontrolled DCD donors (category I: death out of the hospital setting; category II: unsuccessful resuscitation) and controlled DCD donors (category III: awaiting cardiac arrest; category IV: cardiac arrest in brain death) [29]. Due to the growing experience with DCD donation in the last decades, the original Maastricht classification was recently reviewed in order to distinguish the several categories of potential donors in different end-of-life situations, and recommendations for the donation process of DCD donors and the surgical techniques for DCD organ retrieval have been extensively described [30, 31].

Despite the growing experience with DCD grafts in adult LT in recent years, the limitation of the use of DCD livers in paediatric LT recipients might be related to the inferior graft outcomes and higher complication rates reported in adults as well as the scarcity of DCD donors with adequate size-match. In the United Network of Organ Sharing (UNOS) database, the use of paediatric DCD donors represented 0.4% of the whole paediatric donor pool in 1995–2005, this figure increasing to 8–10% in 2006–2016 [1, 19].

According to the current literature, liver grafts from paediatric DCD donors have been allocated to both paediatric and adult recipients. Some studies have included paediatric DCD donors in general analyses of DCD LT that included both adult and child donors, without reporting the specific outcomes of the paediatric grafts [3, 9, 32].

In adult LT recipients, DCD grafts from children have shown similar outcomes to DCD organs from adult donors [33]. The Netherlands group showed that paediatric DCD grafts transplanted into adult recipients have produced comparable results to those obtained with grafts from DBD adult donors in terms of long-term graft and patient survival and are also associated with low incidence of nonanastomotic biliary strictures if the donor warm ischemia time (WIT) was kept short [24].

In the paediatric LT setting, a first series of DCD livers were described by the King's College group in 2006, subsequently updated in 2010, reporting the outcomes of 14 children (Table 1) [17, 18]. After a median follow-up of 3 years, no biliary or vascular complications were observed and patient and graft survival were 100%. The authors commented that the excellent short-to-medium-term outcomes and the low morbidity rate were critically dependent on a careful donor selection (donor WIT < 30 minutes) and meticulous operative techniques (“superrapid technique” retrieval). In addition, the arterial-first reperfusion technique seemed to play an important role in DCD grafts in reducing postreperfusion injury and improving the intraoperative hemodynamics. In this series one DCD graft was split with standard techniques, resulting in one left lateral segment, transplanted uneventfully in a paediatric recipient, and one extended right lobe, which was transplanted after 14 hours of CIT to a 65-year-old male who developed PNF, indicating the importance of shorter CIT in obtaining a good outcome.

In 2006, a UNOS report on DCD paediatric transplantation described the largest experience till then of DCD liver grafts in children [19]. Out of 19 children transplanted with DCD livers, 16 (84.2%) received grafts from DCD paediatric donors and 3 (15.8%) from adult DCD donors. When compared with DBD donors, the incidences of PNF and retransplant were similar and the 5-year graft survival was 79.3% for DCD livers compared to 65.8% for DBD (*p* = 0.3). The authors concluded that paediatric recipients of DCD organs appeared to have results similar to those of patients transplanted with DBD organs, but the small size of the population was insufficient to draw solid conclusions.

Later, the Birmingham group described in different series 7 children transplanted with DCD liver grafts from paediatric and young adult donors [20–22]. In 2009, the first cases of successful use of reduced DCD liver grafts in 2 children with acute liver failure, aged 10 weeks and 6 years, respectively, were described [20]. Both recipients had uneventful early postoperative course with rapid liver function recovery; one patient developed intrahepatic biliary strictures 3 years after transplantation. Despite the small number of cases, this initial report suggested that reduced DCD grafts could be successfully used for urgent paediatric transplantation with the decrease in mortality on the waiting list. Since it is possible that surgical reduction of DCD liver graft could further compromise an already suboptimal graft with the additional cold ischaemia for the benching procedure, the authors pointed out that meticulous donor selection is essential and CIT needs to be kept <8 hours to obtain a good outcome. Subsequently, Gozzini et al. from the same center reported 4 other paediatric DCD LT with no graft or patient loss at 19 months of follow-up, although 2 children developed mild ischemic cholangiopathy, which was treated conservatively [21]. In 2010, a neonate with fulminant acute liver failure secondary to neonatal hemochromatosis was successfully transplanted with an ABO-incompatible reduced-size DCD graft [22]. Following these initial experiences, the Birmingham group defined their criteria to select a suitable DCD donor for paediatric LT recipients which included the following: donor age < 40 years, ITU stay < 5 days, normal liver function tests (LFTs), donor WIT < 30 minutes, CIT < 8 hours, and good appearance and perfusion of the liver [21].

In 2010, Hong et al. from the University of California Los Angeles (UCLA) compared the long-term outcome of 7 children transplanted with DCD paediatric livers to that of 21 children transplanted with DBD paediatric grafts [23]. At 10-year follow-up, no graft or patient loss occurred in either group. Children receiving DCD grafts did not develop PNF, ischemic cholangiopathy, or vascular complications; the development of biliary anastomotic strictures was observed in 1 DCD recipient and in 3 DBD cases. LFTs were similar at 1 week and 3, 6, and 12 months in the two groups. The excellent long-term outcomes and the low biliary complication rate of this single-center experience have been related not only to the adequate donor selection but also to the better regenerative capacity of the biliary tree of young donors. To further reduce the risk of ischemic cholangiopathy, preservation solution was infused* in vivo *into the biliary tree during organ procurement and tissue plasminogen activator was infused into the donor hepatic artery at the back table. These data suggest that the good outcomes were also possibly related to the large experience of the center in adult DCD transplantation, leading to technical refinements of DCD organ retrieval and optimization of logistic planning to minimize the cold ischemia time.

Finally, a recent report from van Rijn et al. described the long-term outcome of 20 paediatric DCD liver grafts, 3 of which (15%) were transplanted in children [24]. In this series, one patient developed hepatic artery thrombosis and died early after transplantation, one developed hepatic artery thrombosis, and another had portal vein thrombosis within one year from LT. Authors commented that the inferior results of this series compared to those previously reported by the Birmingham and UCLA groups might be related to the longer donor WIT (24 minutes versus 14 minutes) and longer CIT (8 hours versus 5-6 hours), which are significant risk factors for graft survival. Moreover, the high rate of vascular complications could be the result of technical issues, but no specific data on recipient surgical procedures were available.

As recently Schlegel et al. [34] reported the utility of the UK-DCD Risk Score, which is an innovative and easy tool permitting identification, prior to organ procurement, of the high-risk DCD LT in adult recipients, we calculated the UK-DCD Risk Score for paediatric DCD recipients in order to define its usefulness in children. However, no paediatric DCD LT study reported data on donor body mass index (BMI) and Paediatric End-Stage Liver Disease score, whose variables are included in the UK-DCD Risk Score. The median UK-DCD Risk Score calculated for the paediatric DCD LT cases reported in the current review (without BMI and PELD/MELD values) is 2 points (range: 2–5), which identify “low-risk” DCD donors for primary nonfunction and ischaemic cholangiopathy. Considering that grafts for children usually come from very small donors (consequently with low BMI) and that the PELD/MELD score often is not correlated with the severity of liver disease in children, the low UK-DCD Risk Score in our series seems realistic, suggesting that DCD donors used for paediatric LT are very “low-risk” donors and extremely selected. In paediatric transplantation, the low UK-DCD Risk Score might be related mainly to the young recipient/donor age and absence of retransplantation in children receiving DCD grafts. Thus, the use of UK-DCD Risk Score could be extremely useful to predict the risk of DCD grafts and to help the careful DCD donor selection for children, but its validation in larger paediatric series is needed.

In summary, 50 DCD liver grafts transplanted in paediatric recipients have been reported so far. The DCD donors were all classified as Maastricht category III with a median donor age of 10 years (0.3 months–64 years) and a median donor WIT of 15 (10–25) minutes. Notably, the donor WIT was defined in 5 reports [20–23, 25] as “the interval between post-withdrawal hypotension (systolic blood pressure < 50 mmHg) and the time of aortic perfusion with preservation solution,” while in 2 studies [18, 26] it was defined as “the interval from withdrawal of life support to initiation of cold organ preservation.” In all DCD paediatric LT the donor “no touch” period was of 5 minutes from the declaration of death.

Except for one (2%) child who died, all recipients were reported to be alive with a median follow-up of 4.7 years (1.7 months–10 years). The retransplantation rate was 6%. A total of 8 (16%) recipients presented postoperative complications including PNF (*n* = 1; 2%), ischemic cholangiopathy (*n* = 3; 6%), anastomotic biliary stricture (*n* = 1; 2%), hepatic artery thrombosis (*n* = 2; 4%), and portal vein thrombosis (*n* = 1; 2%). Despite the small numbers, altogether these findings suggest that children transplanted with DCD livers present satisfactory graft and patient outcomes at medium-to-long-term follow-up, with different features when compared to adult DCD LT. In particular, the incidence of ischemic cholangiopathy seems to be significantly lower in children than in adult DCD recipients, which may reflect the superselective nature of the grafts [35]. Almost all paediatric DCD LT reports suggest that the key points in obtaining good results with DCD liver grafts in children are to ensure a careful donor selection (donor age < 40–45 years, ITU stay < 5 days, normal LFTs, and donor WIT < 30 minutes), limit CIT to <8 hours, optimize logistic planning, and standardize surgical retrieval techniques. Furthermore, these reports cautiously advocate that although satisfactory outcomes of reduced grafts from selected young DCD donors have been reported in small children, further experience is needed before recommending a wider practice of this approach. Finally, the good results of the use of paediatric DCD grafts in adult recipients suggest that paediatric DCD donors may represent not only a source for children awaiting LT, but also for adult candidates.

## 4. Donors Supported by Extracorporeal Membrane Oxygenation in Paediatric Liver Transplantation

Extracorporeal membrane oxygenation (ECMO) is used to provide cardiac and pulmonary support when heart perfusion and lung oxygenation are inadequate to sustain life [36].

The use of ECMO in organ donation has been implemented in the last decades worldwide, and it is currently used in hemodynamically unstable DBD donors or in DCD donors to improve organ preservation for transplantation and to expand the donor pool [37, 38]. After the initial experience of DCD donor support for adult renal transplantation [39], ECMO was also used for liver donors [40, 41]. In adult recipients, several studies showed that DCD donors with ECMO are associated with superior liver and kidney graft short-term function when compared to organs from DCD donors without ECMO support [15, 42–44] with lower rates of hepatic artery thrombosis, PNF, and ischemic cholangiopathy [45].

In paediatrics, the use of grafts from donors supported by ECMO prior to organ procurement has been reported primarily in kidney transplantation [46, 47], while there are limited data for LT. So far, no case of DCD donor supported by ECMO has been reported for paediatric LT, while one case of DBD donor supported by ECMO has been described: in 2013 the case of a 14-year-old DBD donor in whom venoarterial ECMO support was used to treat hemodynamic instability, as a bridge to organ procurement, was reported; the liver was successfully transplanted to a 7-year-old girl who presented normal graft function after 15 months [25].

Recently, Assalino et al. reported two cases of in situ liver splitting in DBD young donors (16 years and 23 years, resp.) in whom refractory cardiac arrest and hemodynamic instability were supported by venoarterial ECMO; after the in situ liver splitting, two children were transplanted with a left lateral segment graft and two adults with an extended-right lobe graft with uneventful postoperative course [26]. Although hemodynamic instability evokes some concern about in situ liver splitting, the authors suggested that ECMO in DBD should not be considered as an absolute contraindication for in situ splitting procedures. The use of ECMO for donor hemodynamic support, enabling in situ liver splitting, could potentially expand the donor pool for paediatric recipients, who are mainly transplanted, at least in Europe, with split grafts. However, this is a very limited experience and further studies are needed to draw definite conclusions.

## 5. Normothermic Liver Machine Perfusion: A Real Future Perspective in Paediatric LT Recipients? 

In recent years, there has been increasing interest in the possible use of liver machine perfusion to minimize the effects of organ preservation and to resuscitate suboptimal donor grafts. Recent trials have shown that normothermic or hypothermic machine perfusion of liver grafts is associated with short-term superiority over cold storage in terms of early biochemical liver function (lower peak serum aspartate aminotransferase), decrease of early graft dysfunction, and reduced rate of discarded organ [13, 14, 48, 49]. The benefits of liver preservation with machine perfusion include also the ability to perform liver viability assessment, extended preservation times, and the potential for liver-directed therapeutic interventions during preservation.

Recently, normothermic machine perfusion (NMP) showed a useful role in reducing ischemia/reperfusion liver damage and in “resuscitating” organs from extended-criteria donors such as DCD [50]. NMP in fact allows continuous graft perfusion with oxygen-carrying red cells at 37° at physiological pressures and flows, providing cell-nutrition and reducing the CIT [13].

The application of extracorporeal liver perfusion in children has not yet been explored probably because paediatric LT recipients are usually transplanted with “optimal grafts” deriving from (a) split livers, which are normally obtained from “ideal donors” (i.e., donor age < 45–50 years, hemodynamic stability, short ITU stay, and normal LFTs) or (b) whole grafts harvested from young size-matched donors. Therefore, the use of machine perfusion to improve the “graft quality” might not be considered necessary in the paediatric transplantation setting.

So far, the largest application of NMP is in DCD grafts, but no cases of DCD livers perfused on NMP have been reported in children. Thus, what is not documented in the literature is the potential paediatric donor offers as DCD grafts, even from very young and small donors, whose use could be increased and safer preservation of these organs could be optimized by machine perfusion.

Recently, there have been some reports on the technical feasibility of splitting during liver machine perfusion [27, 28]. Brockmann et al. reported one splitting procedure during NMP of a graft retrieved from a 56-year-old DCD and declined for steatosis [27]. After 24 hours of liver perfusion on the machine at 37°C, a standard splitting was performed; at the end of the parenchymal division, the left vascular structures (left hepatic artery, left portal vein, and left hepatic vein) were divided and the left lateral segment was separated, while the extended right lobe continued to be perfused on the machine.

Later, four pig livers were split during NMP in order to verify the effectiveness and stability of NMP system [28]. During the splitting procedure the pressures in the portal vein and hepatic artery remained constant and were stabilized by gradually reducing the overall flow. The results indicated that the hemodynamic stability of the liver during the splitting procedure on the machine could be maintained, ensuring a good hepatic microcirculation.

These two reports proved the technical feasibility of splitting during liver machine perfusion and could lead to new perspectives in the application of NMP in paediatric LT. Though not proven by clinical outcomes, these options open up new possibilities for paediatric patients where even marginal liver grafts that are well preserved could be used for small children. How far could we go? Potentially, splitting the liver during NMP could combine the advantages of in situ and ex situ splitting techniques by reducing prolonged CIT and minimizing the ischemia-reperfusion injury to the partial grafts, influencing split liver outcomes [51]. Furthermore, the possibility of assessing organ quality during the NMP could reduce the number of DCD donors declined for liver splitting. Although a successful outcome of transplantation of grafts split during NMP has not yet been demonstrated and more knowledge is needed to assess the real benefits of NMP, the preliminary experiences have definitely provoked enthusiasm.

## 6. Conclusion

Great efforts have been made by the transplant community to expand the donor pool for paediatric LT. The recent advances in transplantation of extended-donor criteria grafts in adult recipients have led to the selective use of these grafts also in children, opening new perspectives for paediatric LT. In the last few years, an increasing number of DCD grafts have been transplanted in children, with satisfactory graft and patient outcomes at medium and long terms and a low incidence of morbidity. A careful donor selection and short donor WIT and CIT are all mandatory for good outcomes of DCD grafts in paediatric candidates, and any additional donor risk factor should be avoided. The use of DCD liver grafts in children could be increased by the donor support with ECMO to optimize the graft quality.

In paediatric LT like for adults, graft preservation on machine perfusion could permit assessing the viability of the organ and reducing the ischemia-reperfusion injury. Moreover, the technical feasibility of splitting liver during NMP could lead to new applications: splitting livers during machine perfusion could increase the number of partial grafts available for paediatric recipients and change the logistics of splitting. The use of new technologies for donor optimization and graft preservation could find selected applications in paediatric LT, but much work still needs to be done.

## Figures and Tables

**Table 1:** Donation after circulatory death in paediatric liver transplantation.

Author	Number of recipients	Recipient age	Recipient gender (M/F)	Type of graft	Donor age (years)	Donor cause of death	ITU donor stay (days)	Donor WIT (minutes)	CIT (hours)	UK DCD Risk-Score^*∗*^	Follow-up	Patient status	Complications
Muiesan et al. 2006 [17] Bartlett et al. 2010 [18]	14	7 yr (8 mo–16 yr)	7 M/0 F	4: whole graft 8: reduced graft 1: split LLS 1: auxiliary LT	23 (10–64)	-7 Anoxia -4 Trauma -3 CVA	5 (2–4)	16 (11–29)	7 (5.5–8.4)	2	41.8 (1.7–74) months	All alive	2: pleural effusion 1: sepsis 4: early ACR 2: ductopenic rejection

Abt et al. 2006 [19]	19	8.7 ± 7.3 yr	12 M/7 F	Whole liver	12.2 ± 14.4	-10 Trauma -7 Anoxia -1 CVA -1 Others	NA	11.6 ± 5.3	8.1 ± 3.6	2	60 months	NA	2: (10.5%) retransplantation 1: (5.3%) PNF

Perera et al. 2009 [20]	2	6 weeks; 7 yr	0 M/2 F	Reduced graft	14; 20	-1 Trauma -1 Meningitis	NA	22; 25	8 h 29 min; 9 h 34 min	5	6 months; 3 years	All alive	1: early ACR 1: ischemic cholangiopathy

Gozzini et al. 2010 [21]	4	9.5 (0.2–17) yr	1 M/3 F	2: reduced graft 2: whole graft	14.2 (11–20)	-2 Trauma -1 Anoxia -1 Meningitis	2.5 (1–5)	12 (10–15)	7 (6–8)	2	19 (8.1–43.4) months	All alive	2: early ACR 2: ischemic cholangiopathy

Gelas et al. 2012 [22]	1	2 weeks	1 M/0 F	ABO-incompatible reduced graft	NA	Anoxia	4	15	6	2	13 months	All Alive	Early ACR; chronic rejection

Hong et al. 2014 [23]	7	28.4 (9.6–59.2) mo	3 M/4 F	Whole graft	2.4 (0.3–6)	NA	<5	24	5 (4–7)	3	10 years	All Alive	1: anastomotic biliary stricture

van Rijn et al. 2017 [24]	3	8.6 (6–13) yr	NA	Whole liver	5 (3–9)	NA	NA	25 (20–31)	8 (7–9)	5	10 years	1 Dead 2 Alive	2: hepatic artery thrombosis 1: portal vein thrombosis

*ACR, acute cellular rejection; F, female; LLS, left lateral segment; LT, liver transplantation; M, male; mo, months; NA, not available; PNF, primary nonfunction; yr, years*. ^*∗*^UK-DCD Risk Score was calculated without body mass index and Paediatric End-Stage Liver Disease score values due to lack of data available.

## Abbreviations

DBD: Donation after brain death
DCD: Donation after circulatory death
CIT: Cold ischemia time
ECMO: Extracorporeal membrane oxygenation
ITU: Intensive therapy unit
LFTs: Liver function tests
LT: Liver transplantation
NMP: Normothermic machine perfusion
PNF: Primary nonfunction
UNOS: United Network of Organ Sharing
WIT: Warm ischemia time.
